# How Well Do Children Understand the Vocabulary of Sleep?

**DOI:** 10.3928/24748307-20190122-01

**Published:** 2019-03-08

**Authors:** Beris Ludwig, Simon S. Smith, Helen Heussler

## Abstract

**Background:**

Sleep surveys, such as the Pediatric Daytime Sleepiness Scale (PDSS), are used to determine a variety of concerns associated with sleep, including excessive daytime sleepiness (hypersomnolence), bedtime sleep behaviors, night awakenings, sleep duration, and sleep-disordered breathing. However, the literacy ability of the patient may not be adequate to ensure comprehension of questions and provision of accurate responses.

**Objective:**

To assess children's understanding of the sleep-associated vocabulary included in the PDSS.

**Methods:**

A cross-sectional, open-response survey was developed for use with students age 4 to 12 years. Prior to completing the instrument, each student was asked the meaning of six key sleep-related words used in the PDSS: drowsy, sleepy, alert, awakened, tired, and awake. The parents/caregivers were requested to record their child's definitions of these key words exactly as stated. Identification of words for “suitable” definitions was undertaken through consultation of three online dictionaries. This enabled the qualitative process associated with open-response surveys to be followed: identification of common themes, chunking of information, and criteria for coding responses. The final sample consisted of word definitions from 325 students (152 boys and 173 girls) from a school enrollment of 727 (45%).

**Key Results:**

A high percentage of children provided “suitable” responses for the words sleepy (84%) and tired (75%). The percentage of “suitable” responses for the words drowsy and awakened gradually increased across the age groups. The words alert and awake were challenging for the children to define, with the sleep-associated definition for alert only being provided by 31% of children overall and awake only being provided by 48% overall. In total, 57% of children were able to provide suitable definitions for at least four words.

**Conclusions:**

Our findings suggest that the results of many sleep surveys using these terms may not yield results that accurately reflect a child's actual state of daytime sleepiness and sleep/wake behaviors. Prior to administering a sleep survey, physicians need to clearly explain the meanings of sleep-associated words used in the survey and thus gain a more accurate reflection of a child's sleep and daytime behaviors. **[*HLRP: Health Literacy Research and Practice*. 2019;3(1):e53–e69.]**

**Plain Language Summary:**

Sleep surveys are used to identify problems with sleep. Children with poor health literacy due to age may not understand the questions and may not provide adequate answers. Children's understanding of sleep-associated vocabulary was assessed using six words: *drowsy*, *sleepy*, *alert*, *awakened*, *tired*, and *awake*. Many of these words were found to be difficult for the children to define.

The completion of a sleep survey by a child or their caregiver is often an integral component of investigations into a child's sleep problems (Kirsch & Chervin, 2011; Wise & Glaze, 2018). This approach presumes that the patient will not have any significant difficulty understanding the language used in the survey, nor any difficulty understanding the required process for completion of the survey. However, the health literacy of the patient may not be adequate to ensure comprehension of questions and provision of accurate responses. Further, the everyday lexicon of health professionals can be specific for particular medical conditions, containing words and phrases not used in conversational English or used with special meaning. Should a child or caregiver's health literacy be inadequate, the accuracy of their responses would be questionable. This could lead to misdiagnoses, misuse of prescribed medication, or even a failure to diagnose at all should the symptoms not be clearly articulated (Yin, 2017; Schreck & Richdale, 2011; National Academies of Sciences, Engineering, and Medicine, 2015). The use of plain language in all health-related communications, both written and oral, is a focus promoted by an increasing number of practitioners (Connelly & Turner, 2017). Surveys are regularly used to determine a variety of concerns associated with sleep, including excessive daytime sleepiness, bedtime sleep behaviors, night awakenings, sleep duration, and sleep disordered breathing (Kothare & Kaleyias, 2008; Tsai, 2010). The Pediatric Daytime Sleepiness Scale (PDSS) (Drake et al., 2003) is designed and used as a self-report measure. It is conventional (and encouraged) in the field to ask the child to report on his or her own sleep-related behaviors and consequential daytime sleepiness (Erwin & Bashore, 2017).

## Background

### International Literacy Levels

International literacy levels in children are rising as a consequence of many countries implementing policies of education for all children. Since 2001, the International Association for the Evaluation of Educational Achievement has assessed reading achievement of children in fourth grade using the Progress in International Reading Literacy Study (PIRLS) process. The most recent PIRLS study, undertaken in 2017, included 50 countries with a total of more than 300,000 students (Mullis & Martin, 2017). Internationally, this assessment of fourth-grade students (most of whom were age 10 years), found that 14% of all children assessed were identified as reading at the low benchmark, and a further 4% below that benchmark (Thomson, Hillman, Schmid, Rodrigues, & Fullarton, 2017). The low benchmark for fourth-grade students states that “when reading and viewing relatively complex Online Informational Texts … students can locate and reproduce explicitly stated information … and begin to make straightforward inferences” (Mullis, Martin, Foy, & Hooper, 2017). These findings for general reading ability suggest that even at age 10 years, approximately 1 in 6 children are unable to adequately comprehend simple texts.

### Health Literacy

Inadequate general literacy skills in a substantial proportion of the population may have particular implications in the health sector. It is recognized that there is a strong correlation between inadequate general literacy skills and poor health literacy (Borzekowski, 2009). The term “health literacy,” first used by Simonds (1974), is used to identify the specific type of literacy required by patients during interactions with health professionals. Earlier definitions of health literacy reflected a more simplistic view, but we now know that literacy is much more than the ability to read health texts (Borzekowski, 2009). Sørensen et al. (2012) reviewed definitions of health literacy and identified six clusters from the 17 definitions included in their literature review. From these clusters, they developed a new and inclusive definition:

“Health literacy is linked to literacy and entails people's knowledge, motivation and competences to access, understand, appraise, and apply health information to make judgments and take decisions in everyday life concerning healthcare, disease prevention and health promotion to maintain or improve quality of life during the life course”

(Sørensen et al., 2012, p. 3).

The complexity of health literacy as a social construct, with the multidimensional aspects as included in the Sørensen et al. (2012) definition, were discussed in detail by Pleasant (2014) who was concerned about how then to appropriately measure such constructs in people. Existing tools have identified that people with inadequate health literacy may have limited comprehension of information (both written and spoken) provided by health professionals or services. Further, they may provide an inadequate medical history and/or relate symptoms poorly, and they may lack treatment compliance (Williams, Davis, Parker, & Weiss, 2002). Comprehension of information through assessment of vocabulary understanding via oral interview has been conducted by Gibbs, Gibbs, and Henrich (1987); and more recently, a similar study using a written multiple-choice format was conducted by Hayes, Dua, Yeung, and Fan (2017). Treatment compliance concerns and medication errors have been identified through various means including patient interview, assessments of prescription labels and supplementary materials, and completion of surveys by health professionals (Yin, 2017). Treatment compliance and health literacy in adults has been assessed using the Test of Functional Health Literacy in Adults (TOFHLA) (Kalichman, Ramachandran, & Catz, 1999). Their findings that years of education and literacy were significantly associated with treatment adherence have been supported in a recent meta-analysis of 48 studies that identified a strong and significant association between levels of health literacy and adherence to treatment (Miller, 2016). Much of the focus of the previous discussion has been on the health literacy of people as patients. However, recommendations from authorities in the health literacy field suggest that the responsibility for gaining an adequate history from a patient, whether an adult or child, and supporting treatment compliance be placed on the clinician (Connelly & Turner, 2017; Paasche-Orlow, Schillinger, Greene, & Wagner, 2006).

### Surveying Pediatric Health Literacy

There is limited research into health literacy in pediatric populations, particularly in children younger than age 12 years. A survey of children's (age 6–12 years) understanding and perceptions of health was undertaken in a primary school in Hong Kong (Fok & Wong, 2002). Using the Delphi technique, they identified that children's understanding of health concepts generally reflected those of the role models in their lives (i.e., parents, teachers, significant others), of advertisers, and of their developmental level. Fok and Wong (2002) did not develop and use a specific instrument to measure children's health literacy. Their interview approach enabled insight into children's understanding of health and also the actions they could take to maintain their health.

Specific measures have been developed for the pediatric population, although some are based on those developed for adults or adolescents. **Table 1** provides an overview of the eight pediatric health literacy measures currently available. Expert reviews by Okan et al. (2018), Guo et al. (2018), and Ormshaw, Paakkari, and Kannas (2013) have assisted in highlighting the necessity of developing validated and reliable measures of health literacy for children and adolescents. Many of these newer health literacy measures are using the work of Nutbeam (2008). He developed a model for health literacy based on his conceptualization that health literacy was generated from two distinct areas: (1) clinical care and (2) personal health control and responsibility. He identified three areas that require research: functional literacy, communicative literacy, and critical literacy (Nutbeam, 2008).

One of the better-known pediatric measures of health literacy is the Newest Vital Sign (NVS). Driessnack, Chung, Perkhounkova, and Hein (2014) explored the possibility of using the NVS, a health literacy assessment instrument used with adults, to provide a quick and simple means of helping providers assess the health literacy of children. They believed that assessment and intervention needed to occur in childhood “before problematic health attitudes and behaviors form and/or take root” (Driessnack et al., 2014, p. 166). In addition, their assessment of health literacy found that parents and children with inadequate health literacy also reported having only 10 or fewer children's books in their homes (Driessnack et al. 2014). This finding further identifies a relationship between low literacy levels and poor health literacy in children. The NVS was further validated by Warsh, Chari, Badaczewski, Hossain, and Sharif (2014). They developed cut-off values in the 7- to 17-year-old age group, and also found that NVS scores correlated to scores on the Gray Silent Reading Test, an instrument that measures a person's silent reading ability.

However, Borzekowski (2009) cautions against using children and adolescents' literacy levels as a determinant of their health literacy. She advocates inclusion of understanding of children's cognitive development in any discussion on health literacy. Wolf et al. (2009) explores the importance of a cognitive skill set, which is more than just skills in reading and numeracy, but something that informs the ongoing development of a child's health literacy. The conceptual model of health learning from Wolf et al. (2009) includes this cognitive skill set and a psychosocial skill set, both of which have numeracy, verbal ability, and reading as common elements. Borzekowski's definition (2009) of health literacy reflects these and the more recent perspectives of the importance of considering a life-course perspective for health literacy:

Health literacy is not just the ability to read health text; rather, it is a set of skills that involve recognizing, processing, integrating, and acting on information from a variety of platforms (p. S284) … Those between the ages of 3 and 18 can seek, comprehend, evaluate, and use health information, especially if materials are presented in ways that are age appropriate, culturally relevant, and socially supported. The development of health literacy among children and young people can empower this vulnerable and “marginalized” group to be more engaged, more productive, and healthier

(p. S288).

A life-course understanding when reflecting on cognitive development has also been emphasized by Allen and Kelly (2015) who state that the “developing mind … [is] astonishingly competent, active, and insightful from a very early age” (p. 88).

In recognizing the significance of the relationship between health literacy and health outcomes as enunciated by Borzekowski (2009), both Connelly and Turner (2017) and Wolf et al. (2009) have identified strategies professionals could readily use when communicating with their young patients. Clearly identifying children's health literacy levels will enhance the development of appropriate interventions. These interventions, in turn, should assist in improving health literacy levels as well as the overall health outcomes for these children.

### Pediatric Health Literacy in Primary Care Settings

The value of adequate health literacy is found in primary care settings associated with a variety of pediatric conditions. Obesity and healthy lifestyle choices are two aspects receiving more attention due to their impact on a wide variety of diseases and conditions. Shih, Liu, Li-Ling, and Osborne (2016) used the Taiwan Child Health Literacy test and body mass index (BMI) data (all children in Taiwan have their weight and height measured every semester) to assess 162,209 sixth-grade students (age 11–12 years). They found that children with higher health literacy were less likely to be obese. A similar association was found by Sharif and Blank (2010) who used the Short Test of Functional Health Literacy (STOFHLA) with 107 children (age 10–16 years). Child STOFHLA score was negatively correlated with child BMI. Reflecting on this data, a specific investigation into food and nutrition literacy may provide further insight into how to develop health literacy to alleviate the obesity epidemic. Doustmohammadian et al. (2017) considered the role of nutrition in development of chronic diseases and used the Nutbeam (2008) model for health literacy to develop a questionnaire for elementary school children (age 10–12 years) in Tehran, Iran. Their food and nutrition literacy scale is a valid and reliable instrument that can be used to assess food and nutrition literacy and promote customized interventions. The authors, however, caution that this tool needs to be used according to social context.

The health literacy of children has been specifically investigated in a number of other areas. Tzeng and Gau (2018) collected 25 pieces of asthma education material for children and interviewed five children (age 8–12 years) about the materials and their usefulness in assisting the children to manage their asthma. Tzeng and Gau (2018) found that most of the education materials were written above the reading ability of school-age children, with only one brochure having a reading level suitable for children in fifth grade or below. Morris, Liabo, Wright, and Fitzpatrick (2007) aimed to develop a questionnaire suitable for children (age 5–15 years) with foot-ankle problems, and Parisod, Axelin, Smed, and Salantera (2016) were interested in developing a deeper understanding on health literacy associated with tobacco use. They interviewed 39 early adolescents (age 10–13 years) and found that there is a variety of factors, both external and internal, that inform the health literacy of these young people. Bollweg, Okan, Bauer, and Pinheiro (2017) and Bollweg, Okan, Pinheiro, and Bauer (2016) developed and validated a self-report questionnaire with fourth-grade students in Germany. They caution, however, that high scores on subjective health literacy measurements do not automatically convert into competence.

### Specific Foci in Health Literacy

Broder et al. (2017) systematically reviewed definitions and models pertaining to health literacy in childhood and youth. Their review identified health literacy as a “multidimensional complex construct” (Broder et al., 2017, p. 1). Broder et al. (2017) found three core constructs when reviewing the 21 models of health literacy: (1) cognitive, (2) behavioral or operational, and (3) affective and conative. Within the cognitive domain, a number of subattributes were identified: knowledge, basic or functional health-related skills, comprehension and understanding, appraisal and evaluation, and critical thinking. This project focused on exploration of the knowledge and the comprehension and understanding attributes. Knowledge, or the vocabulary and a comprehension of the meaning of vocabulary associated with sleep, is fundamental to developing sleep health literacy.

### Sleep Health Literacy

Sleep is one of the specific areas in which surveys are used to gain understanding of the extent of any potential problem. Due to the increasing understanding of the importance of health literacy, survey and program development authors are now addressing the language chosen for these surveys. Bonuck, Schwartz, & Schechter (2016), for example, reviewed their survey drafts with staff and the parent advisory board to ensure clearer language was used in the final version to investigate sleep-related knowledge and sleep problems in early childhood.

Other investigations, albeit with adult populations, have also identified the impact of health literacy on completion of sleep surveys. The Epworth Sleepiness Scale (ESS) (Johns, 1991) has been validated for use in the adult population; however, the language used in the questions was not specifically explored for readability. The concerns about the accuracy of information gained from the ESS facilitated research conducted by Damiani et al. (2013). They identified differences between self-administered surveys and those where the scale is read aloud to a patient, with a significant difference noted in correlation with objective measures such as apnea-hypopnea index and oxygen desaturation index. It was found that physician-administered ESS scores were more highly correlated with these objective measures than self-administered ESS scores. Marra et al. (2018) also found differences between physician- and self-administered ESS scores, with a higher number of compilation errors being found in self-administered surveys. This higher number of errors was also associated with a lower education level of participants. Johns (2015) built on the success of the ESS by modifying the scale for children and adolescents, terming it the Epworth Sleepiness Scale for Children and Adolescents. When presenting this modified scale, he noted that clarification of what is meant by the word “sleepiness” is required. Although the validation report did not specifically mention health literacy, it was stated that the scale is “simple” (Janssen, Phillipson, O'Connor, & Johns, 2017). The health literacy required for completing sleep surveys is unknown, and no specific measure has been developed to expressly assess sleep health literacy. Accessing health care for a sleep problem could, therefore, be challenging should the literacy level of the parents and children be low. Further complexity could occur should the terminology associated with sleep not be well understood.

In Australia, parents of children with sleep disorders currently need to gain a referral from their general practitioner to access specialist assessment and treatment by a sleep physician or an otolaryngologist (ie, ear, nose, and throat specialist). Negotiating this complicated pathway through the health system requires some degree of health literacy (Connelly & Turner, 2017). Once at the specialist, a further degree of health literacy is required, as a comprehensive medical history is typically taken and screening surveys completed. Frequently, the child is also asked to complete a self-report sleepiness scale. There is a possibility that the child's health literacy could affect their understanding of the sleepiness scale, especially if their responses are interpreted by the clinician without a clear understanding of the child's health literacy.

The PDSS (Drake et al., 2003) is an example of a self-report survey commonly used in pediatric sleep-disordered populations. The PDSS was developed initially to measure sleepiness in 11- to 15-year-old children. Drake et al. (2003) did not assess the reliability during the initial development; however, test-retest reliability has been assessed in a number of other language versions including with Brazilian Portuguese (Perez-Chada et al., 2007) and with Chinese (Yang, Huang, & Song, 2010) children and adolescents. When Drake et al. (2003) developed the PDSS, their entire questionnaire contained 32 items. Use of scree plots enabled eight items to be identified within one factor that related to daytime sleepiness. The content validity of the PDSS was, therefore, established during development, although the PDSS has not subsequently been validated against objective measures such as the Multiple Sleep Latency Test. Nixon, Wawruszak, Verginis, and Davey (2006) later evaluated the use of the PDSS in elementary school children (age 5 to 12.9 years) and Vlahandonis, Nixon, Davey, Walter, and Horne (2013) used the PDSS in their initial and 4-year follow-up of a similar age group. Neither study reported on the psychometric properties; however, Nixon et al. (2006) determined that the PDSS was highly correlated with the EDS subscale of the Sleep Disturbance Scale for Children (SDSC), whereas Vlahandonis et al. (2013) reported that they also used the SDSC. None of the studies using the PDSS commented on the sleep vocabulary used within the scale, nor on whether the adults administering the scale gave additional explanations for each question to the children. The vocabulary used in these scales is sleep-specific and possibly adult-oriented. For example, question 1 of the PDSS asks: “How often do you fall asleep or get drowsy during class periods?” (Drake et al., 2003, p.458).

To this point, there has not been an assessment of children's knowledge and understanding of vocabulary associated with sleep. Our study aimed to assess children's understanding of the sleep-associated vocabulary included in the PDSS, because it is uncertain as to what extent younger children comprehend the intent of the questions.

## Method

### Participants

We invited all students (age 4–12 years), their parents/caregivers, and their teachers at a large regional primary school in Queensland, Australia to participate in a study using the PDSS. No child was excluded from participating in this survey because it was deemed important to gain a representative sample from the whole community. Therefore, children with disabilities and children with a variety of cultural backgrounds were included. Although the school population is culturally diverse with a number of children having English as their second language, the staff did not identify any child who would have such limited English that they could not complete the forms. The final sample consisted of word definitions from 325 students (152 boys, 173 girls) from a school enrollment of 727 (45%). Students ranged in age from 4 years and 9 months to 12 years.

### Materials

**Figure 1.**
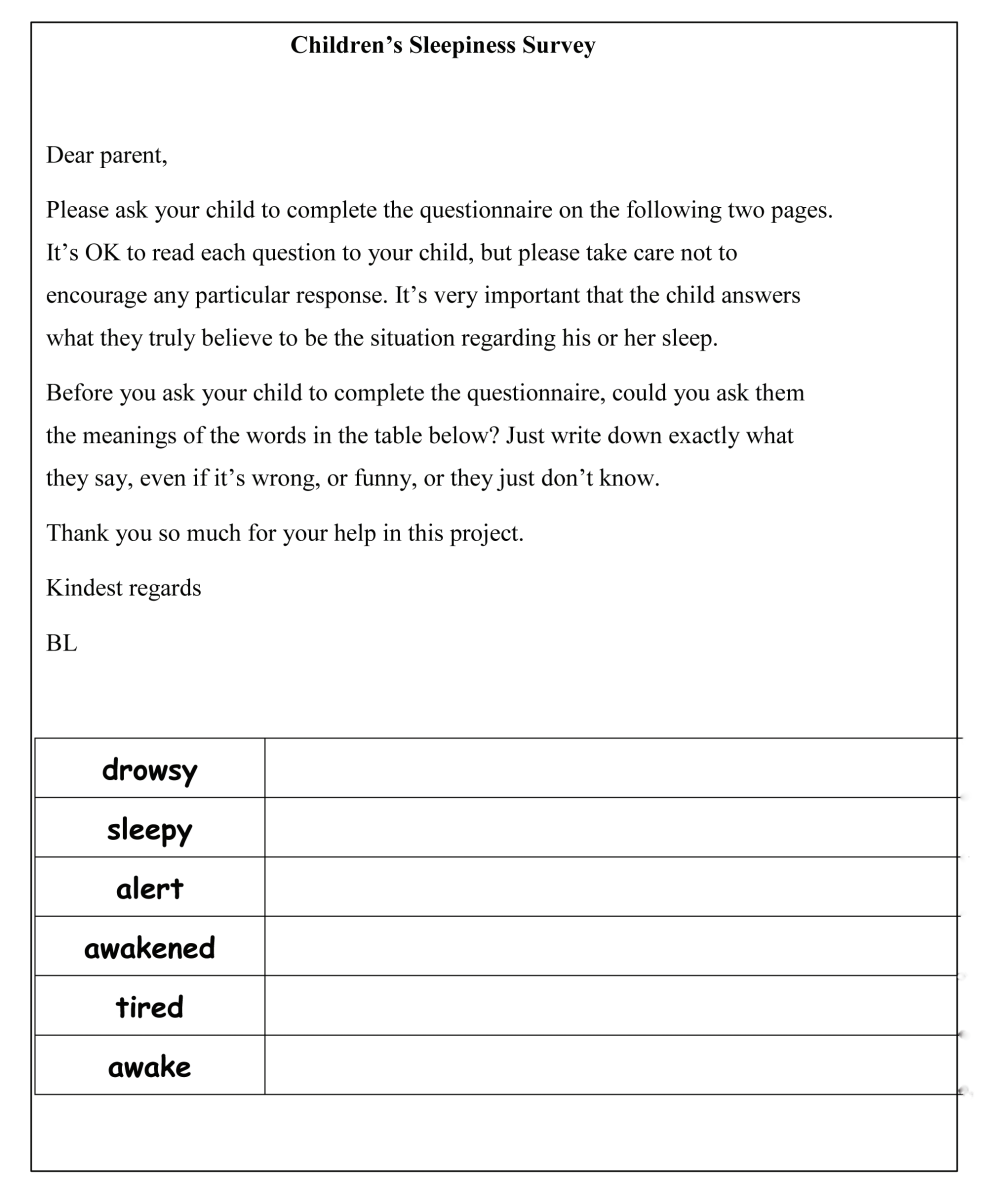
Survey form used to gain children's definitions of sleep-associated vocabulary.

The full survey materials consisted of an introductory letter to the student's parents/caregivers, a simplified introductory letter to the student, a consent form for each, a demographic data form, a form containing instructions and the list of sleep-associated words to be defined, the parent/caregiver version of the PDSS, and a child-friendly modified version of the PDSS. The simplified introductory letter to the students was gauged to be at a grade 3 or grade 4 reading level. Parents were requested to read and explain the letter to younger students. The child-friendly modified version of the PDSS included a facial representation of five stages, from extreme sleepiness to being wide-awake to match each of the five alternatives able to be chosen from (*always* [sleepy] to *never* [sleepy]). The cartoon faces were developed and assessed by Maldonado, Bentley, and Mitchell (2004). They developed a sleepiness scale using faces only to enable diagnosticians, therapists, and researchers a scale that could be used with young or poorly educated people. The faces for the scale were chosen after an intensive process of ranking, validation, and application using 13 groups comprising a total of 835 people. In this study, the faces were used in the children's version of the PDSS to support understanding of the Likert scale alternative responses to each of the eight questions. Prior to commencing the full-scale study in the school, the surveys were pretested with 14 children in a pilot study and used in a parallel study of 10 children with narcolepsy and idiopathic hypersomnia. Verbal feedback from participants in the pilot study and observations by the main researcher (B. L.) of the children in the hypersomnolence study indicated the process was not time-consuming and was readily understood, even with young children and children with low literacy levels. The use of the faces was found to aid comprehension of responses to the questions on the PDSS. A sample of the survey form associated with the data collection for this study can be seen in **Figure 1**.

### Design

This exploratory research study used a cross-sectional, open-response survey to enable an investigation of children's understanding of words associated with sleep. The open-response or open-ended survey method was chosen to gain insight into the sleep health literacy of children in a regional setting. This is a method used for purposes of gaining deeper understanding into the particular area under study (Farrell, 2016). Coding the data was also a consideration, as discussed by Lupia (2018a, 2018b). Although his work is associated with the American National Election Studies, the basic principles of coding appropriately to produce credible and legitimate measures also apply to this survey. Humphrey and Ayers (2018) provide further guidelines for the process of coding the qualitative data, including the importance of identifying key themes for each word and “chunking” the information. To appropriately identify the key theme for each sleep-associated word and provide a process to “chunk” the given definitions, three creditable online dictionaries were consulted (Cambridge Dictionary, 2017; Dictionary.com, 2017; Macmillan Dictionary, 2017). If a given definition contained one of those words/phrases, then it was to be deemed a “suitable response.” The number of “don't know” responses was also counted.

Using dictionary definitions to guide the theme and coding of words was based on the principle that there is an association between vocabulary and comprehension. This association has been identified in descriptive analyses, correlational studies, readability data, and achievement test data (Israel, 2017). Israel (2017) also discusses the concept that a person's vocabulary is also reflective of their knowledge base of a particular subject; therefore, for the purposes of this study, a definition that contained words from one of the dictionaries would reflect a person's understanding of that concept.

In addition to the sleep-associated vocabulary study, this project aimed to use the PDSS to investigate excessive daytime sleepiness in children from their parents/caregiver's observations, self-report, and from their teacher's observations. Correlations among the three perspectives were to be explored (but they are not reported in this article). In addition, demographic data were also to be collected to provide information on the parent's socioeconomic and education attainment levels.

The design of this project ensured no direct interaction between the lead researcher (B. L.) and the participants occurred.

### Procedure

One week prior to distribution of the surveys, the researcher (B. L.) outlined the study rationale and process at a school assembly to which parents had also been invited. The researcher also attended a staff meeting and explained the purpose of the research. At the assembly and at the staff meeting, the researcher stated that assistance to complete the survey would be available should this be required by parents/caregivers. Teachers committed to make themselves available for any parents/caregivers who requested assistance. Students and parents/caregivers were provided with the survey for completion at home.

Prior to completing the PDSS, each student was asked by their parent/caregiver the meaning of six sleep-related words used in the PDSS: *drowsy*, *sleepy*, *alert*, *awakened*, *tired*, and *awake*. The parents/caregivers were requested to record their child's definitions of these key words exactly as stated. Although the data were gathered by parents/caregivers in diverse home environments, they were asked not to encourage any particular response, and for the word meanings to record exactly what their child said, even if wrong, funny, or the meaning was unknown. Demographic information and the PDSS were also completed by the parents/caregivers.

## Results

This large regional school has a diverse student population, with either the mother's or father's highest level of education ranging from Grade 9 to postgraduate tertiary qualifications. Employment information was also captured, and with annual family income ranging from $25,000 (Australian) through to $400,000 (Australian), a wide socioeconomic range range was identified. Data were not collected on the number of single-parent families or on cultural heritage. The school population is culturally diverse, including children from Aboriginal or Torres Strait Islander origin, and is reflective of Australia's history of multiethnic immigration. Some parents sought assistance from school administration staff to complete the survey because English was not their first language.

**Figure 2.**
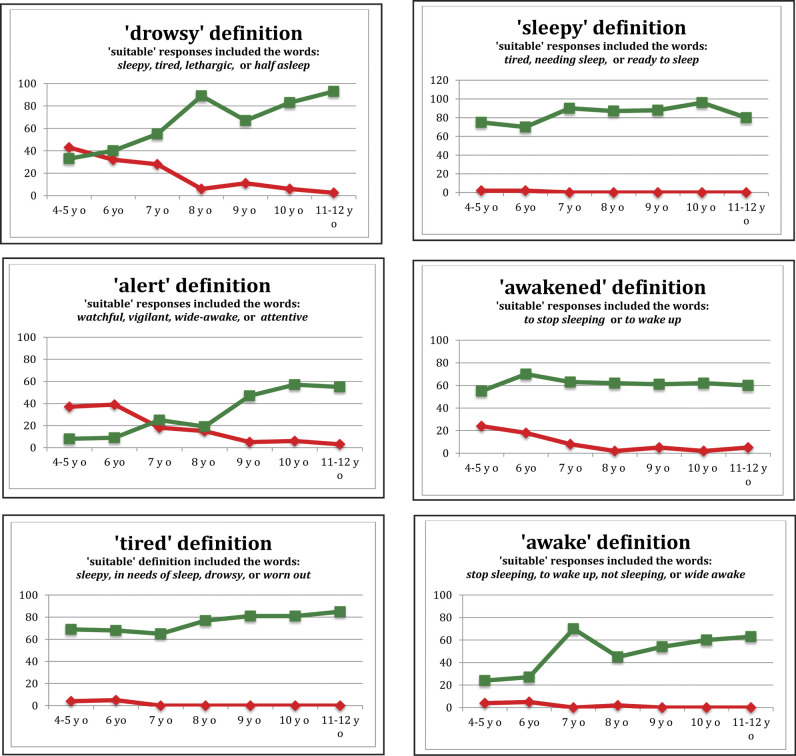
Graphs showing percentages of suitable (green line) responses and don't know (red line) responses for each sleep-associated word in the study. Note that unsuitable responses were not graphed.

The final vocabulary sample consisted of word definitions from 325 children age 4.09 to 12 years (i.e., from preparatory school to Grade 6, which span the primary school year levels in Queensland, Australia.) All definitions were transcribed as text into customized spreadsheets for further analysis. Data were stratified according to the chronological age of the respondents. Use of three online dictionaries (Cambridge, 2017; Dictionary.com, 2017; Macmillan, 2017) yielded formal definitions containing fundamental synonyms for each of the key words (**Figure 2**). A child's definition was considered suitable if it contained one of the key words or key phrases or the intent of understanding was clearly indicated in the form of a short sentence. The number of suitable and unsuitable definitions, and the number of don't know responses, were summed for each word within each age grouping. Both pieces of information were considered reflective of children's expressive vocabulary development. Responses of “don't know” were not counted among the unsuitable responses.

Although the parents/caregivers had been requested not to encourage any particular response and to record word meanings exactly as stated, whether wrong, funny, or the meaning unknown, there is still a possibility of bias. When entering the definitions into the spreadsheet, however, the principal researcher (B. L.) noted that the children themselves, complete with grammatical and spelling errors, wrote the majority of definitions. It is, therefore, assumed that there was no coaching or prompting to promote accuracy of responses.

The percentage of suitable responses for words such as *alert* and *awakened* gradually increased across the age groups; however, the actual percentages were still relatively low (**Figure 2**). The percentage of children who were unable to supply any definition for the words such as *drowsy*, *alert*, and *awakened* gradually decreased across the age groups. *Alert* as a word associated with sleep vocabulary was a challenging word because it is frequently used in connection with emergencies, and this was reflected in the children's responses. By the age of 11 to 12 years, 55% of children were able to generate a suitable definition and only 3% of that age group reported not knowing what the word *alert* meant (**Figure 2**). The word *awakened* generated similar results, with 60% of 11- to 12-year-old children offering a suitable definition and only 5% indicating that they did not know that particular word.

**Figure 3.**
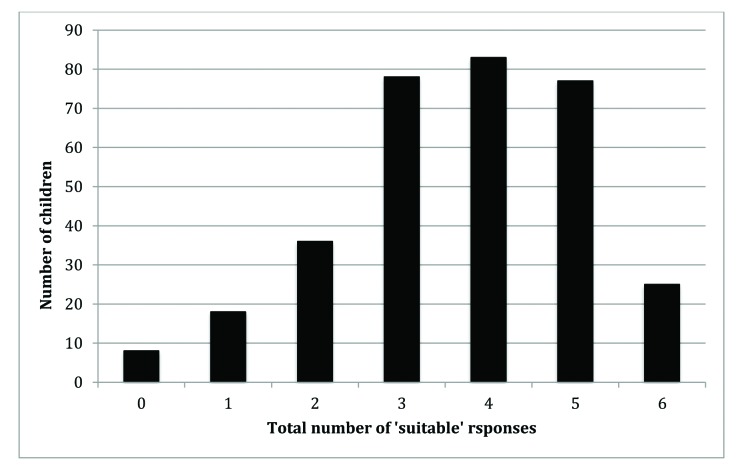
Distribution of overall scores (*N* = 325).

Of the 325 children who provided definitions, eight were unable to give any definition that fulfilled the criteria. **Figure 3** provides an overview of the distribution of scores for each child (ie, the total number of correct definitions for each child). There were 25 children who provided definitions that were considered suitable for all of the six sleep-associated words. Although there were a large number of children who were unable to provide a suitable definition for fewer than one-half of the words, 57% of children were able to provide a suitable definition for at least four words.

The children provided their definitions prior to completing the PDSS. Parents/caregivers and the children's teachers also completed the PDSS. A total of 365 children (44% boys) completed the PDSS, with 24% of those children gaining a score higher than 15, suggesting that they experience excessive daytime sleepiness. The cut-off of higher than 15 was determined by using the work of Meyer et al. (2017). Parent/caregiver (*N* = 352) surveys revealed that 12.5% of their children experienced excessive daytime sleepiness, and teacher surveys on 261 children showed that 8% experienced excessive daytime sleepiness.

## Discussion

This study explored children's understanding of vocabulary used in a widely used sleep survey, the PDSS. A number of other sleep surveys such as the Pediatric Sleep Questionnaire, the ESS, and the Children's Sleep Habits Questionnaire use similar sleep terminology. Results of this study found limited specific understanding of a number of key words associated with sleep and sleep problems, even among children age 11 or 12 years. Sleep surveys are frequently used by professionals in various capacities, such as in research and prior to conducting clinical sleep studies, so as to gain insight into a child's own perception of their hypersomnolence. Sleep surveys use sleep-specific vocabulary that is possibly not in the common lexicon of many children. This study has identified a number of sleep-specific words that are poorly understood by children, although they are found in a number of commonly used sleep surveys such as the PDSS.

The qualitative data yielded perceptions reflective of the developmental expressive vocabulary ability of each child: younger children gave more literal and concrete definitions (**Table A**), often with an example from their own life, whereas older children were more global in their choice of vocabulary to define each word. The oldest groups (age 10–12 years) frequently chose more abstract words when generating their definitions. This is reflective of Piaget's Formal Operational Stage of Cognitive Development, in which children at approximately age 11 years develop the ability to reason in more abstract and logical ways (McLeod, 2018). Borzekowski (2009) suggested that younger children in the preoperation stage would use words associated with “physical appearance or observable action,” whereas older children in the concrete operation stage would use words “associated with particular behaviors and consequences” (p. 285).

Language development occurs quickly in the young brain, from 20 to 50 single words at age 18 to 24 months to more than 2,000 words by the time children commence formal schooling (Nagel, 2012; Parenting Research Centre, 2015). Words such as *sleepy*, *tired*, and *awake* would, therefore, be in common use from a young age. As receptive vocabulary is generally larger than expressive vocabulary, it would be accepted that the children would also understand the meaning of those words. As expected, these words rarely generated a response of don't know. In contrast, however, the accuracy of the definition was not as high as expected, with only 80% of 11- and 12-year-old children being able to give a suitable definition for *sleepy* (**Figure 2**), 85% of 11- and 12-year-old children being able to give a suitable definition for *tired*, and only 63% of 11- and 12-year-old children being able to give a suitable definition for *awake*. These results could suggest that the children may inherently know the meaning of these sleep-associated words but are unable to find alternative words to verbalize their understanding.

The trajectories of suitable word definitions for each of the six sleepiness words surveyed in this study suggest that a significant number of adolescents and adults may struggle to accurately complete sleep surveys or even fully comprehend the information provided to them by a sleep physician or other specialist. These health literacy concerns have implications in a number of areas, such as whether a person understands what it means to be tired or sleepy. Each of those words has differing definitions, generally operational, and each is also challenging to subjectively assess. **Table B** contains a representative sample of the unsuitable definitions of each word according to children's ages.

In addition to the possibility of poor health literacy of the sleep-associated words used in the survey, there was variability of results from the three groups of respondents—parents/caregivers' observations, children's self-report, and teachers' observations. It is hypothesized that the differences in prevalence of excessive daytime sleepiness could be due to the lack of knowledge associated with how sleepy children present. This aspect of health literacy provides a confounding variable. Future research methodology could include education on how children with excessive daytime sleepiness present.

## Study Limitations

A limitation of this study was the possible underrepresentation of families who may have English as their second language. This large, regional primary school has a culturally and linguistically diverse population. Although some of these parents had sought the assistance of staff to complete their surveys, it is probable that a number of parents whose first language was not English may not have been able to read and respond to the invitation to participate. The school population also attracts students from a wide range of socioeconomic backgrounds. Although the demographic data identified representation from a wide range, it is possible that some families from lower socioeconomic status also struggled with reading and understanding the invitation to participate in this study. Alternatively, a major strength of this population-based study was the diversity of respondents, which was reflective of a wide socioeconomic status and culturally diverse background.

## Conclusions

Our findings suggest that the results of many sleep surveys using these terms may not have yielded results that accurately reflected a child's actual state of daytime sleepiness and sleep/wake behaviors. Objective measurements of daytime sleepiness, such as the Multiple Sleep Latency Test or the Maintenance of Wakefulness Test, are used for people with significant sleep problems, generally after a polysomnography (i.e., clinical sleep study) the previous night. The process to gain a referral and then actually undertake a sleep study, however, relies on the use of interview and questionnaires that contain a significant amount of sleep-specific vocabulary.

Paasche-Orlow et al. (2006) have outlined a number of strategies that could be readily included as general practice as clinicians work with their patients to ensure comprehension. Prior to administering a sleep survey, clearly explaining the meanings of sleep-associated words used in the survey may yield a more accurate reflection of a child's sleep and daytime behaviors. Work could also possibly be undertaken when developing new or reviewing current sleep surveys. Words that are more reflective of those used in the common lexicon could be used, or pictorial-based surveys, such as the pictorial ESS (Ghiassi, Murphy, Cummin, & Partridge, 2011) could be further developed. Davis (2017) emphasizes the importance of health care professionals taking the responsibility for ensuring their patients understand all communication, both oral and written. Davis stressed that health literacy should not become the patient's burden. Connelly and Gupta (2017)suggest the practitioner's goal is to use “plain language;” that is, “communication your audience can understand the first time it is read or heard” (p. 40). The onus of ensuring adequate health literacy is on the practitioner rather than on the patient. Bonuck et al. (2016) proposes using the term *sleep health literacy* in the encouragement of healthy sleep habits and when investigating sleep problems. This research supports recommendations that sleep practitioners use appropriate sleep health literacy with their patients, rather than expecting patients to understand the unique vocabulary associated with sleep.

Sleep is essential for good learning outcomes, and should children be sleepy during the day it is important that they have understanding and insight into at least some aspects of their own sleepiness. Consistent with acknowledging child agency, it is necessary that they are asked directly how they feel about their own sleep and possible daytime sleepiness. Therefore, future research is needed to develop a more reliable, validated, child-centric measure with developmentally appropriate language. A more robust measure is required to better address the effects of excessive daytime sleepiness on academic development, behavior, and mental health.

## Figures and Tables

**Table 1:** Pediatric Health Literacy Measurement Instruments

**Author (year)**	**Country**	**Instrument**	**Type**	**Sample Size (Setting)**	**Age Range**
Brown, Teufel, and Birch (2007)	United States (7 states)	KidsHealth KidsPoll	Subjective measurement/self-report	*N* = 1,178 (schools, health education centers)	9–13 years
Davis et al. (2006)	United States	Rapid Estimate of Adult Literacy in Medicine–Teen (adaptation of an existing instrument for adults)	Objective measurement/performance-based assessment	*N* = 1,533 (schools and health care centers)	10–19 years
Driessnack, Chung, Perkhounkova, and Hein (2014)	United States	Newest Vital Sign (adaptation of an existing instrument for adults)	Objective measurement/performance-based assessment	*N* = 47 children (science center) 7–8 y, *n* = 18 9–10 y, *n* = 18 11–12 y, *n* = 11)	7–12 years
Haney (2018)	Turkey	Turkish version of Health Literacy for School-Aged Children scale	Subjective measurement/self-report	*N* = 563 Sixth-graders, *n* = 238; Ninth-graders, *n* = 325)	Mean age range: 13.67 (standard deviation, 1.54 (Sixth graders ∼11 years)
Liao, Liu, Cheng, and Chang (2017)	Taiwan	Taiwan Children's Health Literacy Scale (currently under development and will be based on children's real-life experiences)	Unknown	*N* = 32 children (also interviewed: teachers, *n* = 10; and caregivers, *n* = 11]	11–12 years
Liu et al. (2014)	Taiwan	Child Health Literacy Test	Subjective measurement/self-report	*N* = 162,209	11–12 years
Owens, Spirito, McGuinn, and Nobile (2000)	United States	Sleep Self-Report	Subjective measurement/self-report	*N* = 691	6–11 years
Schmidt et al. (2010)	Germany	New scale: GeKoKids (Health Literacy in School-Aged Children)	Subjective measurement/self-report	*N* = 852	9–13 years
Yu, Yang, Wang, and Zhang (2012)	China	New instrument: Health Literacy Questionnaire	Subjective measurement/self-report	*N* = 8,008 Elementary pupils, *n* = 4,011 Middle school pupils, *n* = 3,997	∼8–13 years

**Table A:** Word Definitions Reflecting Vocabulary Developmental Age

**Word**	**Age**	**Definition**
Drowsy	4- to 5-year-olds 6-year-olds 7-year-olds 8-year-olds 9-year-olds 10-year-olds 11- to 12-year-olds	You're tired You're so tired you can't stay up anymore Sleepy and half awake Not learning in class, tired Kind of sleepy but still awake Feeling like you want to go to sleep Sleepy and lethargic, half asleep
Sleepy	4- to 5-year-olds 6-year-olds 7-year-olds 8-year-olds 9-year-olds 10-year-olds 11- to 12-year-olds	Feeling *[sic]* to sleep When you're so tired that you can't do any work So tired you can't even move You're tired and you want to go to sleep Feel like you are about to fall asleep Needing or ready for sleep Feel very in need of sleep
Alert	4- to 5-year-olds 6-year-olds 7-year-olds 8-year-olds 9-year-olds 10-year-olds 11- to 12-year-olds	Awake Wide awake Means you're paying attention Know what's happening around you Paying attention Able to think clearly, intellectually active Active and responsive
Awakened	4- to 5-year olds 6-year-olds 7-year-olds 8-year-olds 9-year-olds 10-year-olds 11- to 12-year-olds	Not sleeping anymore When someone wakes up A person wakes you up Something wakes you up Been sleeping and someone has shaken you to wake up Awoken by a thing, sound, touch, feel Rouse from sleep; cause to stop sleeping
Tired	4- to 5-year-olds 6-year-olds 7-year-olds 8-year-olds 9-year-olds 10-year-olds 11- to 12-year-olds	Sleepy, yawning When you yawn and you need to have a big sleep Feel sleepy Need to go to sleep When you feel really sleepy Really sleepy and your body is aching Lacking in energy
Awake	4- to 5-year-olds 6-year-olds 7-year-olds 8-year-olds 9-year-olds 10-year-olds 11- to 12-year-olds	You are not sleeping anymore When I wake up Stopped sleeping Finished sleeping – you're awake When you are not asleep Stop sleeping; wake from sleep You are consciously active

**Table B:** Examples of Word Definitions Scored as “Unsuitable”

	**Age (years)**						
**Word**	**4–5**	**6**	**7**	**8**	**9**	**10**	**11–12**
Drowsy	You're drowned I get really angry Starts with a D	When you're a bit angry and a bit sad Two eye lids fighting each other Dramatic	Very sad or very angry So wet When I am about to fall on the floor	Just not feeling up to it – feeling yucky in the morning Not fully awake yet and want to go back to bed Doesn't sleep well	Loopy Grumpy Nausea	Down and falling Angry Had a bad sleep	Not bothered
Sleepy	Your eyes get sore Like a pillow You rub your eyes	Sometimes I feel sick You get sleepy sometimes when you don't sleep at night Feel sleepy when I watch too much TV	When I want my teddy and pillow Lazy Need to go to bed earlier	You do nothing and regain energy Weak	I want to go to bed You haven't had enough rest I just want to lie down and put my head down	That you could go back to bed Sort of like just woke up	Eyes slowly close down and you lose concentration Yawning Just want to lie in bed forever
Alert	I get tired Allergic Sick	I get tired Allergic Sick	When your clock “alert” goes off Freak out Someone trying to hack into your house	Something way amazing is happening Happy If you're cranky	About to fall asleep When the alarm clock wakes you up and you go “arrgh” Someone is in trouble	There is this thing today I need to go to Without sleep Tired but worried about something	Nowhere near tired A bit tired Busy do stuff
Awakened	I get really heavy I want to go and play When people don't need to go to school	The person that was asking was very angry Feel awakened when I have breakfast Awake	So awake you can't even sleep Aahhh Looking around	Eyes opened Ready for a new day Not tired	Oh my gosh, I am late Cold, blankets fall off Wanting to play and walk and chat	Not tired It's a past tense Awake but tired	Annoyed Alert You are conscious
Tired	You're tired You yawn Your body is closing its eyes	No energy Cranky Yawning	When you yawn Closing eyes, lazy Purple under eyes	Weak Frustrated Brain is not working properly	You're doing things in like slow motion and stuff Eyes are closing Miserable	Don't feel like doing stuff You don't feel like getting out of bed No good night's sleep	Half conscious Yawning and restingyour head Not fully opened eyes
Awake	Awake Time to go to school You play games	It means you're ready for school Get up Happy and not angry	You really want to play outside When you jump up and down You are awake	Eyes open You can do stuff You're walking around	Like you're awake and having fun Eyes open You're awake	Busy Moving around That you are awake	Normal everyday concentrated You're awake Ready for most things
